# Until death do us part Adult children’s perspective of their parents’ transition from living at home to moving into a nursing home and the time after death

**DOI:** 10.1186/s12877-021-02633-9

**Published:** 2021-11-27

**Authors:** Christina Bökberg, Jonas Sandberg

**Affiliations:** 1 Department of Health Sciences, Faculty of Medicine, Lund University, P.O. Box 157, SE-221 00 Lund, Sweden; 2 Department of Nursing Science, Sophiahemmet University, Stockholm, Sweden

**Keywords:** Older persons, Home care, Nursing home, Family carer, Next of kin, Transition

## Abstract

**Background:**

Adult children are often key carers of frail older parents providing care for a long period of time in different care contexts. However, research concerning adult children’s caregiving experiences, from providing home-based care to facing the death of a parent in a nursing home is sparse. Thus, the aim was to explore the transition from living at home to moving into and living in a nursing home and the time after death from the perspective of next of kin to an older person.

**Methods:**

A qualitative design using individual interviews with 15 adult children of older persons. The text was analysed using inductive content analysis.

**Results:**

One main category was identified, *until death do us part.* With three generic categories, *living at home, living at a nursing home* and *time after death*, and eight sub-categories. The results describe the transition when an older person lives at home and moves into and lives in a nursing home and the time after death from the perspective of next of kin.

**Conclusion:**

This study highlights many examples of tasks that adult children provide over a long period of time and in different care contexts since they felt that professional care was unable to provide safe and secure care for their older parents. It also highlights the importance for staff to recognize the support that next of kin provide. Furthermore, the study reveal that staff do not offer the relief that they are obligated to provide, to enable next of kin coping with this strenuous transition in life. First after the parent died, there was time for relief since the worrying and the doing of practical things for the parent had stopped.

**Trial registration:**

Current Controlled Trials NCT02708498; date of registration: 26 February 2016.

## Background

Being next of kin to a frail older parent poses many challenges, such as balancing work and family responsibilities and providing the care itself with increased care needs [1]. Additionally, many next of kin experience hardship when a family member moves into a nursing home. This hardship is often related to the loss of daily contact with the family member [2]. Family caregiving builds upon a history of relations between the person in need of care and the family carer [3–5]. Several studies have identified the reconstruction of roles and relations that follows when entering the role of family carer and the caregiving period that follows [6–9]. Studies have shown that it can be a struggle to continue to be the ‘same family as before’ when illness becomes a part of everyday life [10, 11] and when family members face the loss of a mutual future as well as the loss of the image of themselves as a family [12, 13]. To understand the challenges family carers’ face during the process of caring for a family member, transition is used in this paper. Transition is defined as changes occurring in identities, roles, relationships, abilities, and patterns of behaviour [14]. According to Schumacher & Meleis [14], the concept of transition can be useful when exploring the experience of individuals facing periods of instability, such as the imminent death of a parent.

Adult children are key supporters of frail older persons [15], with the provision of instrumental support increasing as their parents age, to the extent that it often impinges on the children’s ability to sustain their own careers [16]. In addition to direct help, adult children also engage in a range of differing types of care, providing supervision and support intended to maintain the dignity and self-esteem of their parents [17, 18]. Children also provide an important link between the parent and the wider society [19] to maintain ‘family connectedness’ [20]. Systematic literature reviews and empirical research indicate that family members’ provision of care plays an important role in the quality of life of older adults [21–23], but the experiences of the family carers during the caregiving period are less described.

It is necessary to appreciate the interdependencies in the caregiving relationship between older persons and their children that develop over time [24]. A temporal model [3] suggests that such interdependencies do not end upon placement in a nursing home; instead, carers enter a new and still involved stage in the caregiving career. Moreover, the placement is not a single event but, rather, a transition that may go on for a substantial period [25]. Considering a move could be initiated by family, friends, and care providers [26] where adult children usually participate in the decision-making process [27]. Previous studies show both active and passive considerations before the placement. Primary reasons include functional and cognitive decline [26, 28, 29], which increases older adults’ needs for both formal and informal care. Deteriorating social and health conditions are main determinants of older peoples’ decisions to move. Specifically, individuals who are older, female, have poor health conditions, have inadequate social support, and lack options for care at home [26, 30]. There are also examples of adult children who are unable or unwilling to care continuously for their dependent parents, deteriorating family relationships due to caregiving burden may lead to adult children relocating their older parents [31].

The time an older person spends in a nursing home is oftentimes the final stage of life since 38% of all deaths in Sweden occur in nursing homes [32]. The final year of life is often associated with symptoms such as pain, depression, confusion, and distress [33, 34]. This situation implies that most of the persons living in nursing homes are in the final stage of life and benefit from a palliative care approach, including care and support for their next of kin. To the best of our knowledge, research concerning the whole care process from home to death from the perspective of next of kin is sparse. Therefore, this paper aims to understand adult children’s caregiving experiences, from providing home-based care to facing the death of a parent in a nursing home.

## Aim

The aim was, from the perspective of adult children to an older person, to explore the transition from living at home to moving into and living in a nursing home until the time after death.

## Methods

### Design and settings

This study had an explorative qualitative design using individual interviews with 15 adult children of older persons. The study is part of the Knowledge-based Palliative Care [in Swedish, KUnskapsbaserad PAlliativ vård, KUPA] project. The KUPA project aims to improve palliative care in nursing homes regarding person-centeredness, quality of life for older persons and their next of kin and the participation of next of kin in the care transition [35]. Approximately 82,400 persons lived in nursing homes in Sweden year 2019**.** These accommodations are mainly offered for older persons (> 65 years) who live in their own homes and their need for care and service became so immense that they can no longer be met in the own home. Access to a nursing home is based upon the older person’s needs, assessed by a social worker in the municipality. Moving into a nursing home usually occurs when the older person is too sick or frail to be able to continue living in their previous home with home care services [36].

### Participants

Inclusion criterion was being an adult child who had had a parent who had been cared for and died in one of the 20 nursing homes included in the KUPA project. One designated contact person at each of the nursing homes asked next of kin whether they would be interested in participating in the project. If they were willing to participate, the contact person delivered their contact information to the research team. Then, the researchers contacted the participants via telephone, providing further information about the project [35].

### Individual interviews

Before the interview started, information about the study was repeated before the written informed consent form was signed. Two interviews with each of the participants were conducted; the first was when the parent was still alive and living in a nursing home, and the second interview was within nine months after the first interview and after the parent had died. Four registered nurses, all females and with experience in palliative and geriatric care, conducted the interviews in the nursing home where the parent had lived.

The first interview focused on the time when the parent still lived at home and the actual situation at the nursing home. The second interview focused on the time between the first interview, the time of death and the current situation after the parent have died. The interview guide consisted of question related to how they coped with the situation at the different stages, their participation in the care, and how the situation impacted on their quality of, life, health, and wellbeing [35]. Follow-up questions were asked to clarify and to gain a deeper understanding. The interviews were digitally recorded and transcribed verbatim and lasted approximately 30–45 min.

### Analyses of the transcribed interviews

The text was analysed using the inductive content analysis described by Elo & Kyngäs [37]. The two different interviews were first analysed separately following the same steps. The transition was analysed started with the authors independently reading the whole interviews repeatedly and making notes and headings in the text, i.e., open coding. Selected units of analysis were then transferred to a coding sheet. During the interpretation of the units, similarities and differences were detected, and codes were created. Codes with similar content were grouped together into eight sub-groups, i.e., abstraction. Then sub-groups with similar contents were grouped together into three generic categories. The generic categories then generated the main category. Both authors separately read and critically reviewed the categories and sub-categories in relation to the interview text. Several combined meetings were held, and the authors reviewed the findings until a consensus was reached and the categories and sub-categories were established.

## Results

The participants consisted of 15 adult children, of whom 13 were daughters and two were sons. The mean age was 64 years (59–68). Most of them, i.e., 12 (80%), were married, two (13%) were widowed, and one (7%) was divorced. Most of the participants, eleven (73%) visited their parent once or more per week. In Table 1 characterises of the next of kin is presented.

**Table 1:** Characteristics of the next of kin

	Relation to parent	Age Year	Civil Status	Frequency of visits
N1	Daughter	65	Married	Once or more per week
N2	Daughter	59	Widow	Sometimes per year
N3	Daughter	71	Widow	Once or more per week
N4	Daughter	51	Married	Every day
N5	Daughter	79	Married	Once or more per week
N6	Son	67	Married	N/A
N7	Daughter	55	Married	Once or more per week
N8	Daughter	68	Married	Once or more per week
N9	Daughter	66	Married	Once or more per week
N10	Daughter	61	Married	Once or more per week
N11	Daughter	63	Unmarried/Divorced	Once or more per week
N12	Daughter	71	Married	Once or more per week
N13	Son	53	Married	Once or more per week
N14	Daughter	61	Married	Once or more per week
N15	Daughter	67	Married	Once or more per month

The analysis of the text resulted in one main category, *until death do us part.* This main category, generated by three generic categories and eight sub-categories, describes the transition when an older person lives at home and moves into and lives in a nursing home and the time after death from the perspective of next of kin. The main category illustrates that family caregiving is an ongoing process containing both emotions and practical responsibilities that continues throughout the whole caregiving career and sometime after death. In Table 2, the main category and the distribution of generic categories and sub-categories are presented.

**Table 2:** Main category, generic categories, and sub-categories

Until death do us part		
Living at home	Living at a nursing home	Time after death
Feeling guilty about one’s shortcomings	Finding out one’s new role	Leaving the caregiving identity
Distrusting formal care	Getting to know the system	Losing close relations
Facing a worsening condition	Dying with dignity	

### Living at home

During the first period in the transition, when the older persons live at home, it becomes obvious that this is a period associated with a gradual increase in worrying, as described in the sub-category *feeling guilty about one’s shortcomings*, and an increase in providing practical things since the next of kin *distrusts formal care.* At the end of this period, the next of kin *face a worsening condition*, and the situation at home is unsustainable.

### Feeling guilty about one’s shortcomings

The next of kin described the time when the parent still lived at home as a period with a constant gnawing worrying related to feelings of unsafety regarding the parent’s well-being. The next of kin worried about what was going on when the parent was alone in the home, such as falls, being unable to find the way back home when going out or forgetting to turn off the stove. Their worrying was related to guilt over not having the time to be present all the time, even if they tried to visit every day. They described that their parent needed to spend many hours on their own, without anyone to socialize with. The home care staffs did not seem to have the time to sit down for a chat; they just rushed in and out doing their tasks.

*Emotionally, it's very hard… you have her in your head the whole time (N4)*

Another issue related to the increasing guilt and worrying of the next of kin was their parent’s intake of food. One example of this worry is that the home care staff delivered a box with (cold) food but did not have the time to stay or to chat during the meal or even to help with feeding. This situation implied that the parent did not eat and just put the food box in the refrigerator. To improve the situation and make the parent eat, the next of kin said that they cooked for their parent and stayed with them during the meal.

*It was terrible the way she had it... They came morning, noon and night and threw a meal box on the table and she didn't touch it. I guess she didn't want to sit there by herself... and cold it was, the food... When we were there, we fed her and helped her… and we brought food from home. Yes, then she ate when we sat with her* (N5)

### Distrusting formal care

The next of kin expressed that they did not trust the formal care provided in the parent’s home, because they experienced it as fragmented. Examples of fragmentation were the lack of continuity in the care, which implied many different care providers coming to the home, and care regularly provided by young care providers with no education. This fragmentation made the next of kin feel that the care and services provided in their parent’s home were unsafe and implied that the next of kin felt obligated to provide many practical things for their parent. For instance, they cooked, cleaned, washed, shopped, took care of finances, arranged transport, and they called the parent daily to check that everything was okay. The next of kin were exhausted due to the heavy burden of taking care of their parent. They also reflected on the ‘age in place ideology’ resulting in a decrease in nursing home places and implied that care and services were provided longer in the home. They could not understand how this arrangement could be cheaper since they perceived the home care providers driving back and forth to take care of many people and there seems to be a lack of organization. Looking back on this period, some next of kin reflected that after their parent moved into the nursing home, they felt much more secure that their parent was properly taken care of. Then, the level of worrying and the tiredness decreased.

*You're supposed to live at home. it's to save money because it's cheaper but I find it so hard to understand that. How it can be cheaper for people to whizz around and then poorly organized... where mom lived... there were five different ones who came… then they would go away in different places and edges* (N8)

### Facing a worsening condition

As time went on, the parent’s care needs increased, their condition worsened, and the next of kin experienced the situation at home as unsustainable. The move from home to the nursing home was described by the next of kin as going back and forth in emotions with periods of feeling well and feelings associated with guilt, worrying, anxiety and depression.

The transition of leaving the home and moving into the nursing home could be initiated by either the next of kin or the parent. This had an impact on the transition. The transition went more smoothly if the move was initiated by the parent compared to being initiated by the next of kin. When the initiative came from the next of kin, the struggle to move their parent to the nursing home was harder. Then, the next of kin needed to persuade both their parent and the social worker about the advantages of leaving the home and moving into a nursing home. The persuasion was described by the next of kin as a balancing act between the parent’s autonomy and their safety and well-being.

*I cleaned and washed and, yes, everything, but in the end, I realized that I cannot manage it anymore… Yes, it was not the easiest thing to make him want to move in. There was great strife, yes, there was* (N15)

The next of kin described a critical incident as the last straw and as the final cause of leaving the home and move into the nursing home. This critical incident was by some next of kin defined as a fall with or without a fracture, followed by hospital care. Then, it was obvious that the parent could no longer continue living at home.

*my mother wasn't ready for it, but then after her 90th birthday she fell and couldn't stand on her feet anymore then she realized she couldn't live at home anymore* (N8)

### Living at the nursing home

In the second period in the transition, the older person had moved into the nursing home, and then, the next of kin needed to *find out their new role* and *get to know the system.* This was complicated by the fact that they did not receive requested information and perceived the staff as lacking the proper competence and as being too few. This situation implied that the next of kin felt that they needed to supervise the care provided. As the end of life of the parent came closer, the next of kin expressed that it was important that the parent was *dying with dignity*, meaning that the parent should remain at the nursing home with highly familiar staff offering symptom relief.

### Finding out ones’ new role

The next of kin felt welcome at the nursing home and felt that the staff were kind to them, offering them food and beverages when visiting. However, the time just after the parent had moved into the nursing home felt unclear for the next of kin. Requests for introductions and information about routines at the nursing home were expressed by the next of kin. The lack of information made them feel insecure regarding their new role and their responsibilities to their parent in relation to the staff. Some next of kin expressed that the staff was not interested in their knowledge about the parent and their preferences and routines. The next of kin described that the staff collected written information about the parent but that this information ended up in a binder that was never or seldom read.

*Once she's arrived here, it's they [the staff] who are in charge of her and not me… I'm the one who's her voice. I know her so well, we have always socialized a lot, so it is a bit strange that they cannot listen a little bit anyway… I would like them to sit down and tell me what it means and what desires they have for me… i.e., how it is set up* (N10)

The next of kin wanted to be involved in the care, and asked for information, preferably on a regular basis. For them to take part in the care, lack of information was a major obstacle. To obtain information, the next of kin needed to contact several staff members, registered nurses, and managers. They thought that the staff should be obligated to inform them of changes in their parent’s condition or if a critical incident occurred. However, that was not the case.

*We want, regardless of the situation, that at least once a month, they contact one of us and tell us what they know or see… but this was mainly on our initiative. I didn't like it* (N1)

The next of kin expressed that if they were too insistent in their contact with the staff or if they made too many demands, the staff would ignore them, which in turn could affect their parent. Therefore, it was a matter of balancing participating and withdrawing their actions. However, because of the lack of information and the sense of insecurity regarding their responsibilities, the next of kin felt they needed to control and/or supervise the care. One daughter (N2) expressed it as follows:

*We did not get any insight into her medical papers, so when I was there and visited, I went and snooped in the binders. I had to see the big picture, and I took her medical papers… and copied them and did research at the National Board of Health and Welfare*

Not only was information delivery between the next of kin and the staff described as lacking. The next of kin reflected on the organization at the nursing home and found that it was fragmented and did not work smoothly. In their descriptions, communication and information delivery between staff members, registered nurses, physicians, and managers failed, which meant that the next of kin needed to ask many different staff members for information concerning their parent. Contact persons were described as a good source for continuity and for the next of kin to obtain information. However, they were often replaced.

*It was mostly there when the communication was lacking, between the staff and the nurse. They changed the contact person as well, and this was several times really, and they did not manage it quite like I think they should have* (N1)

### Getting to know the system

The next of kin described the staff as kind and as showing a great deal of engagement, patience, and empathy, doing their very best. Staff members who had long experience increased the sense of security for the next of kin. However, the next of kin also described that the number of staff members and their competence were too low and that the staff turnover was immense. The shortage of staff was described as most obvious for the next of kin not only during evenings and weekends but also during holiday periods, when the number of new and unexperienced staff members increased. The next of kin who had their parent at the nursing home for a long period of time described a decrease in the number of staff members over the years.

*Everything is safe when those who are old in the game are working. With all the new staff, I understand that it must be like that when someone gets sick. Then, it has to be new staff and whatnot, but there is a big difference. The very, very best is when those who know the job work and know the person and do not back away from the hard stuff, and I say nothing about the young people because everyone of course must learn somewhere, but there is a big difference* (N14)

The perceived shortage of staff had consequences for the parent’s well-being, as noted by the next of kin. The next of kin exemplified this by older persons needed to go to bed early in the evening and stay in bed until after breakfast. This in turn led to older persons becoming immobilized and/or catching pneumonia according to the next of kin. Furthermore, they needed to wait for help, were unable to go out or could not have company.

The next of kin expressed that they continued performing practical tasks after their parent moved into the nursing home; however, such tasks decreased. Instead, they took great responsibility for the medical care of their parent, such as medication. When the next of kin noticed that their parent was (negatively) affected by a medication, they contacted the registered nurses and physicians. If medical staff did not listen to the next of kin, they acted on their own by taking the pills away from their parent and even taking their parent out of the nursing home.

*… I asked the nurse to pick it [drug] out. Then, she says, "No, I cannot do so." Then, I took mom down to the cottage, and I removed that drug myself* (N4)

Another example of medical tasks provided by next of kin was when the parent was ill or caught an infection. Once again, they contacted registered nurses and physicians and asked for a medical check-up, for example, taking blood samples and measuring the temperature or blood pressure. The next of kin felt a great sense of responsibility concerning their parent’s care, implying that they took actions when the staff were passive or lacked knowledge or when they felt more knowledgeable than the staff.

*…So, mom was sick on a Friday, and I know that Saturday and Sunday nothing happens… she had a strong urinary tract infection, and she just got sicker and sicker and worse and worse, and in the end, she stopped talking because she gets so affected, and then, I ask them, "Please, can you ask for or take a blood sample?", and* ***then,*** *the nurse listened and asked the doctors to do so, and she had a high CRP, so then, she was hospitalized* (N2)

### Dying with dignity

For the next of kin, it was important that the life of their parent was dignified, and they perceived the imminent death of their parent as a natural transition. Thus, it was not important for them to prolong their parent’s life if doing so became associated with pain or unconsciousness. When the next of kin were asked about palliative care and the breakpoint conversation [when patients and families are informed about changing focus from curative to palliative care in accordance with Swedish national guidelines], they replied that they were unfamiliar with the concept and that no one had initiated such a conversation. However, the next of kin expressed that they wished the dying transition of the parent to be short, preferably occurring during sleep.

*…Just mom may stay and die here in her bed… I don't want her to be sent away and… a lot pumped in and kept going on and so, absolutely not, I would rather she may die here in peace and quiet… in her bed… and I don't feel that her life needs to be extended in any way. She’s been so sick… I think, make her life stop now. Because she has nothing, that is wrong to say, a good life, for she is well in the small life, in her own little world. That's where she's happy. But I can see she doesn't have a life, so to speak… it would be just as nice now if, if she does not get better again, for what does she have in front of her? Then, it's better that she goes to sleep [dies]…* (N11)

The next of kin did not want to transfer their parents to hospital care at the final stage of life. Instead, they preferred that the parent stay at the nursing home treated with medication against pain and anxiety and be surrounded by staff who were well known to the parent.

The next of kin described the dying process as starting with a critical incident, such as a fall or an infection, with or without hospital care. Then, the parent had difficulties rehabilitating to the previous status. The dying process could also start with the parent ceasing to eat and drink, as described by the next of kin. Then, the dying process became smoother, with a gradual decrease in functions and ending with a peaceful sleep.

*It will of course be a slow deterioration and transition, and in the end, she is no more… it is of course so natural, more natural does not exist in any way* (N1)

The next of kin described that the staff tried to relieve their anxiety and stress during the parent’s dying process by making frequent calls when the status of the parent changed, offering them drinks and beverages, offering possibilities to stay over at the nursing home when they were tired and needed to rest.

*But the staff were very understanding… I didn't expect it, professionally. No, I didn't… not the concern for us as well (N5)*

### Time after death

The third and last period, when the parent had died, was time for the next of kin to *leave the caregiving identity.* This period was related to not only grief and loss of the parent but also relief. Since the worrying and the doing of practical things for the parent had stopped, they had plenty of time to spend on their own. During this period, they reflected on *losing close relations.* The next of kin narrated that during the transition, they had close relations with both their parent and siblings. However, the close relations with their parent gradually decreased during the transition compared to the relations with their siblings, which decreased rather quickly after the parent died.

### Leaving the caregiving identity

For the next of kin, the grief started before their parent died since the parent was described as altered during the long period of time before death. The time after death was related to grief and loss of the parent, but feelings of relief were also expressed by the next of kin. They described their parent’s very last period in life as being associated with suffering, combined with expressed wishes for life to end. Therefore, the grief period was rather short. Nonetheless, the next of kin said that a smell or a song could act as triggers to remind them of their parent; then, they were overwhelmed with feelings and surprised by their reaction.

*Of course, it is empty when mom is not here any longer, but since she was so sick lately… it was nice, she did not want to live… so, it was nice as well, once she was gone* (N8)

The next of kin were content with the efforts they made for their parent for a long period of time and said that they have no regrets and that they could not have done anything else/more.

*I feel like I've done exactly what I've been able to do. I haven't been able to do more for mom* (N1)

However, guilt was expressed by those next of kin who were not present at the point of death and by those who did not understand how sick their parent was.

*The blame is that I was not there when he died (N9)*

The next of kin described that lack of nursing home beds and the long queue implied that they had a week to empty the room after their parent died. This situation was stressful for them. They were grieving, and at the same time, they were supposed to make decisions regarding what items to keep and what to throw away. The staff tried to make the situation easier for the next of kin, if possible, by not only offering a longer period to empty the room but also offering small things of care, such as coffee.

*We only had seven days to empty the room because there are desperately few places and there are long queues, so quickly after someone has died, it is important to get a new person in as soon as possible (*N8)

The next of kin were pleased to be able to follow the instructions given by their parent before death about how to arrange the funeral. The death was perceived as a natural process after a long life. Then, the funeral and/or memorial service were perceived as bright, bringing family and friends together to remember the parent.

*She wanted to be cremated, and she wanted a white wooden coffin with pink flowers. It was like her wish… "That's all I want, and then, you can do whatever you want." So, when she was cremated, we decided that we would scatter her ashes in the archipelago. So, we gathered all the family on the rocks and had such a nice day. We sat there and told some stories about her, and we had photographs of her when she was young, and we talked, me and my brother, about our memories, and everyone got to say something, and then, we put flowers in the water and… yes, it was beautiful* (N1)

Most of the next of kin described that after the death of their parent, they had plenty of time to spend on their own, making it possible to take up previous interests, socializing and travel, while other next of kin expressed extreme tiredness and difficulties concentrating.

*I have felt a bit tired and more unfocused than I have felt before. There’s been very, very, very much happening… So, sometimes I've felt a little more tired than I usually do* (N1)

### Losing close relations

When the next of kin looked back and reflected on the whole transition, they described how the relationship to the parent had changed. The time when the parent was still living at home was a period associated with a great deal of anxiety for them since they were always thinking about their parent’s well-being and worrying whether any critical event had happened at home. The next of kin wanted to be present and close to their parent and refused to go abroad or perform activities of their own. Once the parent moved into the nursing home, their anxiety decreased, and it was possible for them to do things on their own. After the parent passed away, the next of kin described a sense of freedom in which they had plenty of time on their own, but looking back, they missed not having the practical things to do.

*It sounds cruel maybe, but it's a bit of a feeling of freedom* (N7)

The next of kin to parents with dementia described that the person they used to know gradually disappeared during the transition or was gone before death occurred. This was illustrated by lines such as ‘it was no longer my mother,’ ‘she was like a child,’ and ‘I became a mother to my mother’. This affected the relationship and was described by the next of kin as something that made it difficult to talk to their parent, implying that they just sat quietly together. The next of kin also described feelings of frustration and anger during the transition when it was no longer possible to discuss and communicate with their parent. The next of kin said that in the past, they used to ask their parent for advice or discussed difficult situations, problems, and dilemmas. When this was no longer possible, the conversation instead became rather superficial.

*I did not tell her because it just made her worried. She often was told only positive things. It was so difficult to find topics of conversation, so we socialized a lot. It's no longer mother, you miss it a little bit* (N14)

The next of kin also brought up conflicts in the past that had not been properly discussed or solved. Now, doing so was no longer possible. Some of the next of kin could accept the situation, while others found it harder, regretting that they did not have the discussion earlier. The next of kin tried to protect their parents from worrying by not telling them things that might upset them.

*…Then, I attack her… with harsh words, unfortunately. I regret today that I was so hard. I could have been a little nicer or whatever. I can feel guilty about that… and then there are things that maybe we should have talked about a long time ago, but we have not done that, and it is not possible to do so now. Because she would just be sad and not understand what I mean, and it makes no sense* (N11)

The next of kin also reflected on the relationship with their siblings during the transition. This relationship was described as important for them. During the transition, the siblings turned to each other for support, they took turns visiting the parent, and they passed on information to each other. They described that they had shared the responsibility for the parent.

*We support each other well. There was always someone who could help… And if I couldn't, my sister or brother could… it was nice that you have one or two to share it with. It feels very positive. Yes, you simply share the responsibility… When you have good relationships with your siblings. It has worked really well* (N10)

Those next of kin who did not have any sibling perceived taking responsibility on their own as lonely and missed having someone to talk with.

*I often say it's hard to be an only child… I had no one to share it with* (N13)

After the parent had died, the next of kin described that the relationship with their siblings had changed. The tight communication between them had stopped, and the topic of discussion changed.

*You don't have quite as much to discuss anymore because that was what it was all about… mom, health care and all that, the whole piece… We talk about everything else as well, but this care for her somehow tied us together* (N5)

## Discussion

The findings of the present study further illustrate the complex, subtle and often implicit interactions that lie behind and influence the transition from living at home to moving into and living in a nursing home and the time after death from the perspective of next of kin. The identified categories show how both the illness trajectory, the care responsibilities, the place of care and the intimate relationships between a next of kin and a parent are linked close together and influence the life for the next of kin. In other words, this transition is seen to operate at several levels and to have different levels of influence on all the major actors involved.

The findings suggest that next of kin focus the greater part of their efforts on trying to ensure continuity and maintain their relationship with their parents, but as the sub-category ‘*feeling guilty about one’s shortcomings*’ shows, they feel a constant gnawing worrying regarding their parent’s well-being. At the same time, they want to ensure that their parent’s needs are effectively being met by staff, but they described distrust in formal care. Thus, there is a need to appreciate the interdependencies in the caregiving relationship between older parents and their next of kin that develop over time [11]. It is apparent that caring for a frail old parent is a transition that may last for a substantial period. Informal caregiving generates a range of mixed emotions, and many carers require support, but support related to the emotional impact of placement is often not provided [7, 16, 18].

The difficulties in planning for entry into a nursing home – both physically, but more importantly, the emotionally difficulties – featured prominently in the study and is further elaborated in the generic category ‘*living at the nursing home’*, which describes the largely ambivalent emotional responses to the move and the difficulties experienced in initiating and sustaining roles and relationships with staff after the move. This is particularly expressed by the next of kin in the sub-categories ‘*finding out one’s new role’* and ‘*getting to know the system*’, where the next of kin experienced not receiving adequate information from the nursing home staff and expressed that the number of nursing home staff members and their competence were too low. This situation implied that the next of kin felt that they needed to supervise the care provided.

It has been suggested that entry into a nursing home results in a reduction in stress for carers [38], but the findings from this study does not fully support this assumption. Family carers describe feelings associated with guilt, worrying, anxiety and depression. Which is in line with research showing that the transition and post-placement period can instead be an extremely stressful experience [39–42]. To reduce stress, the transition to a nursing home should be eased by promoting an understanding of relocation as a normal phase in the caregiving trajectory [11]. This means recognizing that the problems and worries experienced by families do not disappear when the person who is being cared for moves into long-term care [11]. Therefore, while there may well be a sense of relief, next of kin still experience a range of negative feelings, including sadness, grief, loneliness, anger, and guilt, with few sources of support [25].

Following the placement of a close relative in a nursing home, many families remain actively involved in caregiving [43, 44] and continue to visit regularly [45]. However, the findings of this study indicate that next of kin might face a host of uncertainties about which new roles to adopt and how to interact with staff. Studies show that there has often been little contact with the nursing home prior to the placement, and consequently, expectations and role responsibilities have rarely been discussed [46, 47]. This can result in misunderstandings between relatives and staff [40], with interactions often being far from ideal [48]. Carers might experience difficulties in questioning standards of care and in making suggestions for change, as they are afraid to appear critical [49]. The role of adequate preparation for next of kin is a central issue in the findings, and it is important to enhance practice to meet their needs at this difficult time. Staff must have the skills to address the ambivalent feelings that next of kin experience following the decision that the move is necessary. Next of kin must be better prepared not only for the move but also to find their new roles to integrate with the nursing home.

Following placement, the next of kin described uncertainty about how to find their role in their parent’s new home. They felt that they were unsure how to manage the insight that the death of the parent was imminent. The findings also showed that the next of kin knew that death was a natural transition, but at the same time – even if the dementia diagnosis and the illness trajectory was described as a loss of a parent – the actual death was something that marked the end of a life -long relation, an end of the identity as a caregiver and loss of a parent. The emotions related to these changes show the need for staff to help next of kin prepare for the separation from their parent. This should be an integral and important part of the entire transition of admission into nursing homes and subsequent adjustment [18, 50]. It is essential that staff not only recognize the importance of their interactions with relatives but also have the time, skills, and inclination to help next of kin prepare for the imminent death.

From the findings of this study, one of the most important emotional consequences for next of kin following their parent’s move into a nursing home is the loss of the parent and their feelings of shortcomings and loneliness, a conclusion reinforced in other studies [20, 24]. None of the next of kin in the present study were prepared for such feelings, and their emotions were not sufficiently acknowledged by professionals, even if there were examples of appreciation for the next of kin by staff, such as the offer of a cup of coffee. Overall, this study suggests a need for a better preparation for separation [6] and preparation for loss [2]. This would be facilitated by greater recognition of carers’ emotional needs.

What was quite apparent from this study is the importance that carers attach to continuity of care, that is, their continued involvement, in one form or another, in the life of their parent. This primarily related to the attempts made by next of kin to reconstruct their relationship with their parent, with next of kin working hard to sustain the individuality and ‘personhood’ of their parent by maintaining those aspects of life that they enjoyed and that in some way marked them as unique. The next of kin described the transition from living at home to moving into a nursing home and the time after death as difficult, and it generated a range of mixed emotions. Next of kin require support and practical support was given in some ways. However, planned support related to the emotional impact of the placement and the imminent death of the parent was not provided. Instead, support was given by staff based on personal initiative.

The next of kin’s mixed emotions and experiences of nursing home involvement suggest that next of kin expect to have an active part in their parent’s care. Promoting family engagement fosters a sense of partnership and involves the exchange of experiences, expectations, and values. Adopting a person-centred approach and sharing information and decision making with family caregivers [51] can contribute to the quality of care experienced by relatives. This result points to the families’ desire for emotionally sensitive care and not just for technically competent performance of tasks. Supporting family caregivers through times of transition as described in this paper is an important function, and the concept transition might help in understanding the impact of life changes. The findings from this study may be helpful in for staff in community care as well as in nursing homes to consider family carers’ journey through the transition, and in explaining the range of experiences that may be encountered.

By using Schumacher & Meleis’s concept of transition [14], when exploring the experience of periods of instability, such as facing the imminent death of a parent, we can better understand caregiving experiences – from providing home-based care to facing the death of a parent in a nursing home.

### Limitations

One way of increasing credibility in this study was selecting a heterogeneous sample to obtain variation in the experiences of the participants, thereby highlighting several different perspectives on the phenomenon being studied. The selection of participants contributed to variation in experiences regarding being a next of kin to an older person in the transition from living at home to moving into a nursing home and the time after death. This study sought to achieve transferability, which has been facilitated by the reader being offered a clear description of the study participants, the selection of the participants and the context of the study. However, the study included only adult children, which can give a biased picture compared to the picture that might have been obtained if spouses had been selected; this issue may constitute a weakness of the study. All participants had to respond to the same interview guide, which strengthens the study’s dependability. The length of the answers to specific questions differed between the participants. Some participants spoke longer in response to questions related to situations that were difficult for them, while other participants were shorter in their replies. It appears the participants had a need to talk about these experiences and appreciated being listened to. Thus, it can be regarded as a strength that the results of this study are based on narratives that the participants voluntarily chose to share. By generously presenting quotes, the reader is given the opportunity to review the categories. In this study, quotations within each category and sub-category are presented to facilitate the reader’s assessment of the reliability of the study’s results. Another strength is that all interviews were conducted by the same interviewers and that the data collection period was relatively short (nine months). Furthermore, the authors’ prior knowledge of what was studied was not considered to be a disadvantage; rather, it contributed different perspectives and angles during the work of analysis.

## Conclusions

The findings of this currant study suggest that being an adult child to an older parent during the transition, from living at home to moving into and living in a nursing home and the time after death, was a transition associated with a gradual increase in worrying and providing practical things. This transition was complicated by that the adult children did not feel they receive enough support or information about routines at the nursing home as well as changes in the parent medical status from staff. Instead, they felt that they needed to supervise the care provided and sometimes take actions to prevent critical accidents. Finally, after the parent died, there was time for relief since the worrying and the doing of practical things for the parent had stopped.

This study provides many examples of tasks that adult children perform over a long period of time and in different care contexts because they felt that professional care was unable to provide safe and secure care for their parents. This study highlights the importance for staff of recognizing the support that adult children provide to frail parents and offering relief so that next of kin can cope with this strenuous transition in life.

## Data Availability

The datasets generated and/or analysed during the current study are not publicly available, even though the data are anonymized, the study contains sufficient details to enable the identification of individuals. Therefore, before approving the study, the Regional Ethics Review Board in Lund set restrictions regarding the accessibility of the data. Data are available from the KUPA project leader Professor Gerd Ahlström gerd.ahlstrom@med.lu.se upon reasonable request.

## Abbreviations

KUPA project: Knowledge-based Palliative Care project
